# Up-Regulation of microRNA-126 May Contribute to Pathogenesis of Ulcerative Colitis via Regulating NF-kappaB Inhibitor IκBα

**DOI:** 10.1371/journal.pone.0052782

**Published:** 2012-12-28

**Authors:** Xiao Feng, Hao Wang, Shicai Ye, Jiaxi Guan, Wenkai Tan, Si Cheng, Guoli Wei, Weiyun Wu, Feng Wu, Yu Zhou

**Affiliations:** 1 Department of Gastroenterology, The Affiliated Hospital of Guangdong Medical College, Zhanjiang, Guangdong, People’s Republic of China; 2 Department of Medicine, University of Chicago, Chicago, Illinois, United States of America; University of Florida, United States of America

## Abstract

**Background:**

MicroRNAs (miRNAs) are important post-transcriptional regulators. Altered expression of miRNAs has recently demonstrated association with human ulcerative colitis (UC). In this study, we attempted to elucidate the roles of miR-126 in the pathogenesis of UC.

**Methods:**

Expression of miR-126, miR-21, miR-375 and the potential targets NF-κB inhibitor alpha (IκBα, IKBA or NFKBIA), Polo-like kinase 2 (PLK2) and v-Crk sarcoma virus CT10 oncogene homolog (CRK) were assessed in 52 colonic biopsies from patients with active UC, inactive UC, irritable bowel syndrome (IBS) and from healthy subjects by quantitative RT-PCR and immunofluorescence analyses. Regulation of gene expression by miR-126 was assessed using luciferase reporter construct assays and specific miRNA mimic transfection.

**Results:**

We found that the expression of miR-126 and miR-21 were significantly increased in active UC group compared to the inactive UC, IBS and healthy control groups (*P*<0.05). In contrast, the expression of IKBA mRNA and protein was remarkably decreased in the active UC group compared with the other three groups (*P*<0.05). The expression of miR-126 and IKBA mRNA were inversely correlated in active UC patients (*P*<0.05). However the expression of miR-375, PLK2 and CRK showed no difference between each group. Furthermore, we demonstrate that endogenous miR-126 and exogenous miR-126 mimic can inhibit IκBα expression. Finally, mutating the miR-126 binding site of the IKBA 3′-UTR reporter construct restored reporter gene expression.

**Conclusion:**

miR-126 may play roles in UC inflammatory activity by down-regulating the expression of IKBA, an important inhibitor of NF-κB signaling pathway.

## Introduction

Ulcerative colitis (UC) is one of two major types of inflammatory bowel diseases (IBD) and is presented as a chronic, relapsing and remitting inflammatory disease limited to the mucosal layer of the rectum and colon. An increasing incidence of UC has recently been reported worldwide in the last 20 years, especially in China [1]. Pathogenesis of IBD is not yet fully understood. It is currently proposed that the pathogenesis of IBD involves genetic susceptibility variation, inappropriate immuno-response to microbes in the gut and undetermined environmental factors. Furthermore, pathogenesis of UC may vary in different ethnic populations and demographic regions. Genome-wide association studies have identified multiple susceptibility loci with high risk of IBD development [2]–[5]. Gene expression studies found a significant set of genes differentially expressed in patients with UC compared to healthy individuals [6]–[9]. However, it is still unclear how such differential expression of genes is regulated.

microRNAs (miRNAs) have recently been recognized as important post transcriptional regulators. Mature miRNAs are a class of small, non-coding RNA molecules in length of 20–25 nucleotides(nt). miRNAs bind to sequences located in the 3′-untranslated region (3′-UTR) of target mRNAs in the RNA-induced silencing complex and regulate mRNA degradation or repress their translation. miRNAs have been proven to play critical roles in many biological processes such as cell differentiation, proliferation, apoptosis and tumorigenesis [10]–[11]. Mounting evidence has shown that miRNAs are critically involved in the regulation of inflammatory and immune responses. Inflammatory cytokines and microbial components (flagellin and lipopolysaccharide, LPS) induce expression of miRNAs such as miR-146a, miR-155 and miR-132, via the transcription factor nuclear factor-kappaB (NF-κB) pathway in myeloid cells [12], [13]. It has been shown that miR-146a modulates Toll like receptors (TLR) and tumor necrosis factor-α (TNF-α) signaling pathways through a negative feedback loop, by reducing expression of Interleukin-1 receptor-associated kinase 1 (IRAK1), TNF receptor-associated factor 6 (TRAF6) and cyclooxygenase-2 [13]–[15]. miRNAs including miR-155 and miR-150 are shown to influence the fate of immune cells, and to regulate adaptive immune response such as antigen presentation and T-cell receptor signaling [12], [16], [17].

An interesting study first demonstrated the association of 11 miRNAs with active UC [18]. Since then, several research groups worldwide reported that various miRNAs are differentially expressed in colonic tissues of UC and Crohn’s disease (CD), as well as in blood and stool samples from IBD patients [19]–[27].

The genome-wide microarray screening study identified a group of miRNAs differentially expressed in active UC in the American population, including 3 downregulated miRNAs (miR-192, miR-375 and miR-422b) and 8 unregulated miRNAs (miR-16, miR-21, miR-23a, miR-24, miR-29a, miR-126, miR-195 and Let-7f) in the colons of active UC patients [18]. However, the association of miR-126 with human IBD has not been further investigated.

In this study, we aimed to characterize the roles of miR-126 in the pathogenesis of human UC. We first found that miR-126 was significantly increased in the colons of active UC patients compared to other control groups. We also found that among three potential targets of miR-126, nuclear factor-kappaB inhibitor alpha (IκBα, also named as IKBA, NFKBIA) was decreased and inversely correlated with miR-126 expression in colons with active UC. Furthermore, we demonstrated that endogenous miR-126 and exogenous miR-126 mimic can inhibit IκBα expression. Our findings suggest that miR-126 may play an important role in the regulation of inflammation in chronic inflammatory disease, including UC.

## Materials and Methods

### Human Tissues

Colonoscopic pinch biopsies from the sigmoid colon of 52 patients with chronic active UC, chronic inactive UC, irritable bowel syndrome (IBS), and normal, healthy patients undergoing screening colonoscopies were obtained from The Affiliated Hospital of Guangdong Medical College, Zhanjiang, China. Biopsies from all patients were assessed between September 2008 and December 2009 and the clinical characteristics of the patients are indicated in Table 1. The diagnoses of active UC and inactive UC were confirmed by colonoscopy and pathological findings. Disease activities were determined using the Ulcerative Colitis Disease Activity Index (UCDAI) [28], which is a series of qualifiers about the symptoms of ulcerative colitis including stool frequency, rectal bleeding, appearance of the lining of the colon, and a physician rating of disease activity. Each of these items was given a number from 0 to 3, with 3 being the highest for rating of disease activity. Ratings of 0–2 were recorded as remission, 3–6 as mild, 7–10 as moderate,>10 as severe UC respectively. Patients with UCDAI >8 were included in the study.

**Table 1:** Clinical Characteristics of Patients.

	Control	Active UC	Inactive UC	IBS
No. of patients	15	12	10	15
No. of Male, (%)	7(46.7%)	5(41.7%)	4(40%)	9(60%)
Age(yrs)				
Mean±SE	47.1±14.1	48.8±21.3	37±12.4	40.2±9.9
Range	30∼70	17∼80	25∼58	27∼60
DAI score				
Mean±SE	–	8.91±0.9	0.9±0.88	–
Range	–	8–10	0–2	–
Diease duration(yrs)				
Mean±SE	–	1.8±1.0	4±1.2	3±1.6
Range		0.5–4	2–6	1–6

### Isolation of Total RNA

Colonoscopic biopsies or cultured cells were rapidly transferred into 1.0 ml of TRIzol reagent (Invitrogen, Carlsbad, CA) and total RNA was extracted according to the manufacturer’s instructions and stored at −80°C until used.

### Real-time Quantitative Reverse Transcriptase-polymerase Chain Reaction (qRT-PCR)

The NCode™ VILO™ miRNA cDNA Synthesis Kit(Invitrogen, Carlsbad, CA)and ImProm-II™ Reverse Transcription System(Promege, Madison, USA)were used to reverse-transcribe the miRNA and mRNA, respectively. For qRT-PCR on biopsy tissues, 500 ng of total RNA was converted to cDNA. 2 µl of cDNA from each sample was amplified by real-time fluorescent qPCR, using SYBR Premix Ex Taq™ (Takara Bio, Inc., Tokyo, Japan). The expression of each target miRNA in tissues was calculated relative to U6B, a ubiquitously expressed small nuclear RNA used as internal control. Expression of beta-actin was used as normalization control in mRNA qPCR. For miRNA qPCR, the reverse primer was the NCode miRNA universal qPCR primer (Invitrogen, Carlsbad, CA). Forward miRNA primers and mRNA primers were compound by Sangon Biotech Co, Ltd (Shanghai, China) and are listed in Table 2. A comparative threshold cycle method was used to compare each condition with controls.

**Table 2:** Primers Used for Quantitative Real-time PCR in This Study.

Name	Direction	Primer (5′-3′)
For microRNA qPCR		
Universal qPCR primer	Reverse	NCode™ VILO™ miRNA cDNA Synthesis Kit (Invitrogen)
miR-126	Forward	TCGTACCGTGAGTAATAATGCG
miR-21	Forward	TAGCTTATCAGACTGATGTTGA
miR-375	Forward	CGGCTCGCGTGAAAAA
U6 snRNA	Forward	CTCGCTTCGGCAGCACA
For mRNA qPCR		
IKBA	Forward	CACTCCATCCTGAAGGCTACCAA
	Reverse	AAGGGCAGTCCGGCCATTA
PLK2	Forward	GAACACCCGCAGTAGAAAACA
	Reverse	GACCCACTGAAATGATGTGCT
CRK	Forward	AGGGTTATCCAGAAGCGAGTC
	Reverse	CTTCCCACTGACCACTCACAT
Beta-actin	Forward	GGCGGCAACACCATGTACCCT
	Reverse	AGGGGCCGGACTCGTCATACT

### Immunofluorescence

Immunofluorescencet staining was performed according to standard protocol (Santa Cruz Biotechnology). Briefly, a 5-µm frozen section from each case was fixed in acetone at 4°C for 10 min and blocked with 10% goat serum albumin at room temperature (RT) for 30 min. the sections were incubated with rabbit anti-human IκBα primary antibody (1∶400) (Santa Cruz Biotechnology) overnight at 4°C. After rinsing in phosphate-buffered saline (PBS) for three times, the sections were incubated with secondary polyclonal Cy3 labeled goat anti-rabbit-594 IgG (1∶400) (Zhongshan Goldenbridge Biotechnology. Beijing, China) for one hour at RT in dark, and then washed with PBS. PBS replaced anti-IκBα antibody as a negative control. 4′,6-diamidino-2-phenylindole (DAPI) was used to counter-stain the nuclei for 5 min at RT. The staining was evaluated on a Leica converted fluorescence microscope and the fluorescence intensity was measured using Image-Pro Plus 6.0 software. We quantified the total Cy3-fluorescence intensity (as shown in red) of the mucosa and submucosal layers as representation of protein expression level of IκBα. Intensity of DAPI staining (blue) was used as an internal normalization control for adjusting fluorescence signals among different slides.

### Identification of Potential Downstream Targets of miR-126

To predict potential targets of miR-126, three in silico analysis programs were used for microRNA targets prediction, including MicroCosm Targets (http://www.ebi.ac.uk/enright-srv/microcosm/htdocs/targets/v5/), Targetscan (http://www.targetscan.org/) and PicTar (http://pictar.mdc-berlin.de/).

### IKBA 3′-UTR Wild Type and Mutant Constructs

The 3′-UTR of IKBA mRNA (RefSeq NM-020529) has a putative miRNA-126 binding site. In order to test the effects of miR-126 on the expression of IκBα, we created a dual-luciferase reporter construct containing two replicate miR-126 binding sequences corresponding to 1502–1538 nucleotides of IKBA mRNA sequence using an established method [29]. Briefly, the wild type sequence (5′-TGTATTGTTGGTAATTATTTTGGTACTT TTATGATGTTGTATTGTTGGTAATTATTTTGGTACTTTTATGATGT-3′) was created by PCR with forward primer IKBA-XhoIF (5′-CCGCTCGAGTGTATTGTTGGTAATTATTTTG GTACTTTTATGATGTTGTATTGTTGG-3′) and reverse primer IKBA-NotIR (5′-ATAAGAA TGCGGCCGCACATCATAAAAGTACCAAAATAA-TTACCAACAATACAACATCATAAAAG-3′). To generate a construct containing the miR-126 binding site mutants, we used a forward primer (Mut IKBA XhoIF: 5′-CCGCTCGAGTGTATTGTTGGTAATTATTTACCAT GTTTTATGATGTTGTATTGTTGG-3′) and a reverse primer (Mut IKBA NotIR: 5′-ATAAG AATGCGGCCGCACATCATAAAACATGGTAAATAATTACCAACAATACAACATCATAAAAC-3′) bearing six substituted nucleotides corresponding to the 5′-seeding region of the miR-126 binding site (shown as underlined in wild type sequence). The PCR parameters were as the followings: initial denature at 94°C for 5 min, 30 cycles of 30 sec at 94°C, 30 sec at 55°C and 20 sec at 72°C, and a final extension of 5 min at 72°C.

The wild type and mutant sequences were then cloned into the XhoI and NotI sites downstream of the Renilla luciferase gene of the dual-luciferase report vector psiCHECK-2 (Promega Corporation, Madison, USA) according to manufacturer’s protocols. The wild type and the mutant constructs were named as psi-IKBA and psi-mutIKBA, respectively. The sequences of the constructs were verified by DNA sequencing. The constructs were transformed in E.coli JM109 and the plasmid DNA was purified using TIANpure MidiPrep kit (Tiangen Biotech, Beijing, China).

### Construct Transfection and Luciferase Report Assay

Human colonic cancer cell line HT29 and HCT116 (Beijing Institute For Cancer Research, China) were maintained in DMEM containing 10% FBS at 37°C in a 5% CO_2_ incubator. HT29 cells were seeded in 24-well plates overnight before transfections. Luciferase report constructs (100–250 ng/well) were transfected into cells using Lipofectamine 2000 (Invitrogen, Carlsbad, CA) according to the manufacturer’s protocols. Cells were harvested at 24 hours after transfection. Renilla and Firefly luciferase activites were measured using the Dual Luciferase Reporter Assay System (Promega Corporation, Madison, USA) according to the manufacturer’s instructions. Experiments were performed in quadruplicate wells and datas are represents of three independent experiments.

### miRNA Mimic Transfection

HT29 cells were seeded into 24-well plates at 90% confluence and kept in a 37°C and 5% CO_2_ incubator overnight. Varying amounts of miR-126 mimic or negative control mimic (RiBoBio, Guangzhou, China) were transfected into HT29 cells using Lipofectamine 2000. The transfected cells were incubated at 37°C in a 5% CO_2_ incubator for 24 hours. Cellular total RNA and protein were harvested separately, and stored in −80°C until used.

To determine the specific effect of miR-126 on 3′-UTR of IKBA, the miR-126 mimic and the psi-IKBA luciferase construct were co-transfected into HT29 cells using Lipofectamine 2000. Renilla and Firefly luciferase levels were measured at 24 hours post-transfection using the Dual Luciferase Reporter Assay System. Experiments were performed in quadruplicate wells and datas are represents of three independent experiments.

### Western Blot

Western blot was performed according to standard protocol. Briefly, 15 µg protein per samples were separated by sodium dodecyl sulfate-polyacrylamide gel electrophoresis (SDS-PAGE) and then transferred to polyvinylidene fluoride (PVDF) membrane. The membrane was probed with rabbit anti-human IκBα primary antibody (1∶1000 dilution. SC-371. Santa Cruz Biotechnology, Santa Cruz, CA, USA) overnight at 4°C. The membrane was then incubated with HRP-labeled Goat Anti-Rabbit IgG (1∶1000. Beyotime, Jiangsu, China) at RT for one hour. Mouse anti-human Tubulin antibody was used to detect tubulin as a loading control. The integral of optical density of bands was measured using Quantity One image analysis software (Bio-Red, Hercules, CA, USA).

### Statistical Analysis

Experimental results are expressed as mean values ± standard error. Statistical analysis for qRT-PCR and Immunofluorescence was performed with 1-way ANOVA for comparing all pairs of groups. The correlation between miR-126 and IKBA mRNA was calculated according to Pearson correlation analysis using SPSS software, v13.0 (International Business Machines Corporation). *P*<0.05 was considered significant.

### Ethical Statement

The study was approved by Institutional Review Board of the Affiliated Hospital of Guangdong Medical College. All patients gave written informed consent for their participation.

## Results

### Differential Expression of miR-126, miR-21 and miR-375 in UC Tissues

A genome-wide microarray screening indicated that miR-126 and miR-21 are increased and miR-375 is decreased in sigmoid colons of active UC patients [18]. To determine whether miR-126, miR-21, and miR-375 are differentially expressed in UC patients from different ethnic groups, we analyzed the expression of these candidate miRNA in Chinese patients with active UC.

**Figure 1:**
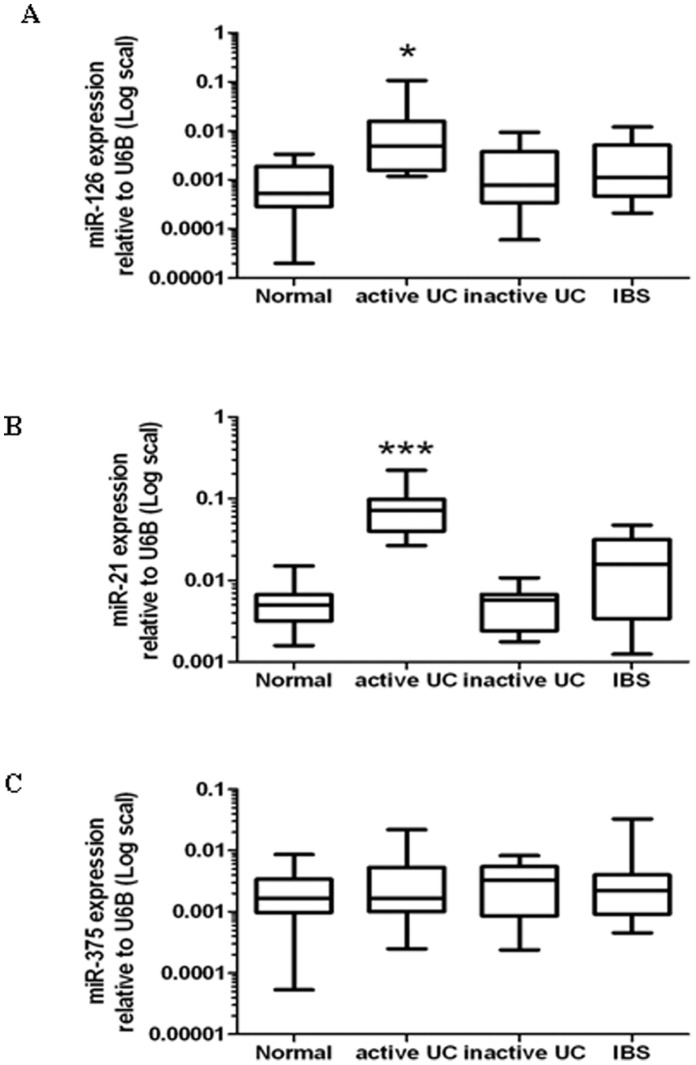
miRNAs expression in sigmoid colonic tissues. Expression of (A) miR-126, (B) miR-21 and (C) miR-375 were measured by quantitative RT-PCR in RNA samples of active UC tissues (n = 12), inactive UC samples (n = 10), IBS samples (n = 15) and healthy controls (n = 15), respectively. Data is presented as box-whisker plots (box - 25%–75%, whisker - 5%–95%; line - median). (*P<0.05, ***P<0.001, compared to that of normal healthy individuals).

A total of 52 sigmoid colon pinch biopsies from patients with histologically confirmed active UC, inactive UC, IBS and normal healthy control subjects were obtained. Clinical characteristics of each patient group are listed in Table 1. We performed quantitative real-time PCR on total RNAs isolated from these biopsies. We found that expressions of miR-126 and miR-21 were significantly increased in active UC tissues compared to healthy control tissues (Figure 1 A and B). The expression of miR-126 was increased 18-fold (*P*<0.05), while the expression of miR-21 exhibited a 14.7-fold increase (*P*<0.05), in active UC tissues compared to healthy controls, respectively. However, expressions of miR-126 and miR-21 were not significant changed in either inactive UC or IBS tissues compared to healthy controls (both *P*>0.05 respectively) (Figure 1). The results are consistent with findings by previous microarray analysis [18]. However, there was no statistical significant difference in expression of miR-375 among the sample groups tested (*P*>0.05) (Figure 1C).

### Selection of Potential Downstream Targets of miR-126

In order to identify downstream targets of miR-126, we used three miRNA targets prediction programs including MicroCosm Targets, Targetscan and PicTar. In silico analysis indicated approximately 900 mRNA targets of miR-126. Among these targets, Polo-like kinase 2 (PLK2, a tumor suppressor) and v-Crk sarcoma virus CT10 oncogene homolog (CRK) were shown as targets of miR-126 in previous studies [30]–[33]. However, there currently is no evidence that PLK2 and CRK are association with UC.

**Figure 2:**
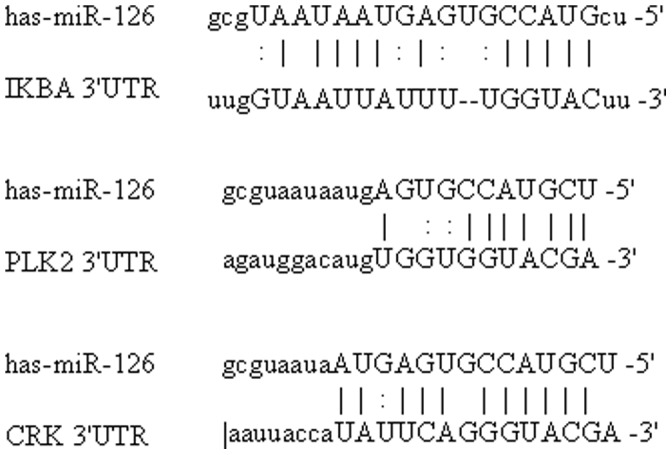
Sequence alignments between miR-126 and target mRNAs. Base pairing complement suggested the putative miR-126 binding position at 445–465 of 3′-UTR of the IKBA mRNA sequence.

Interestingly, IκBα, an inhibitor of NF-κB pathway, is also one of the predicted targets of miR-126. A putative miR-126 binding site that encompasses 11 perfectly matched nucleotides was defined at the 3′-UTR (un-translated region) of IKBA (Figure 2).

In an effort to delineate potential downstream targets and further understand the mechanisms of miR-126 over-expression for pathogenesis of UC, we selected three putative targets (IKBA, PLK2, CRK) for further molecular and functional analyses.

### Both IKBA mRNA and Protein Decreased in Active UC Tissues

**Figure 3:**
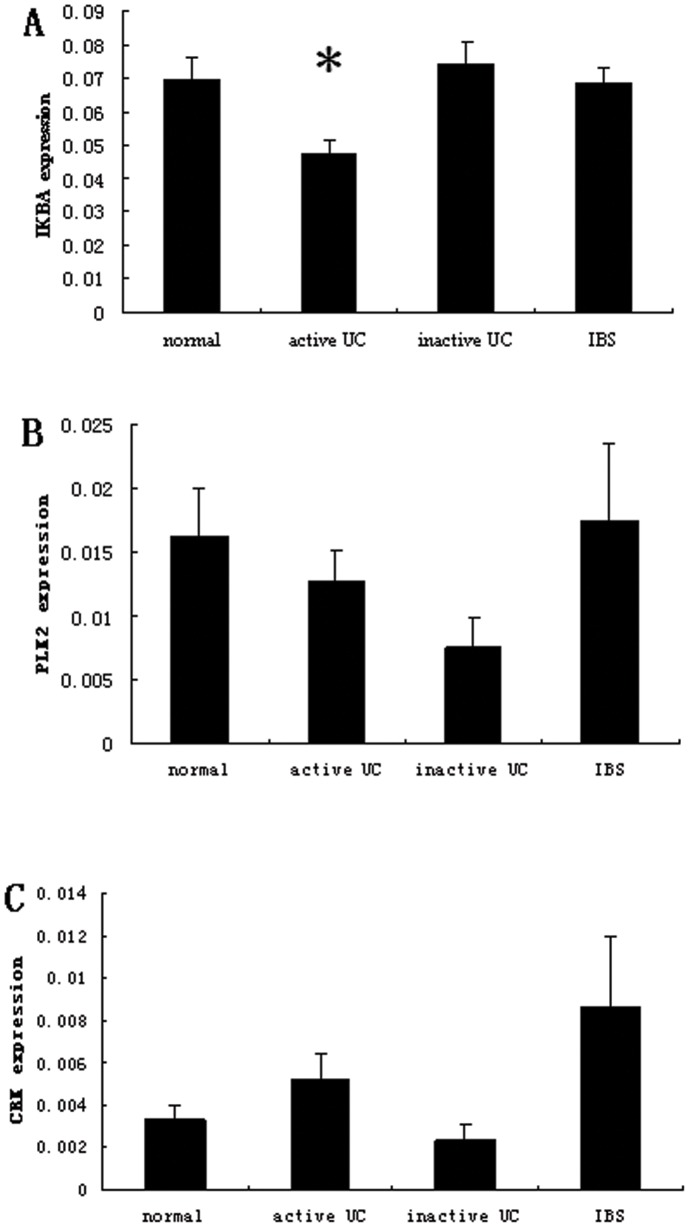
IKBA, PLK2 and CRK mRNA expression in sigmoid colonic tissues. Expression of (A) IKBA, (B) PLK2 and (C) CRK were measured by quantitative RT-PCR in RNA samples as indicated in Figure 1. Data is presented as mean ± SEM (*P<0.05 compared to that of normal healthy individuals).

To experimentally identify the targets of miR-126 in colon tissue, we performed qRT-PCR analysis on the expression of IKBA, PLK2, and CRK in total RNA from the same 52 sigmoid colon pinch biopsies. Among the three candidates, we found that only IKBA mRNA was markedly decreased in active UC tissues compared to healthy controls. A 32% decrease in IKBA mRNA expression was observed in active UC tissues relative to healthy controls (*P*<0.05) (Figure 3A). However, IκBα expression in either inactive UC or IBS tissues was not significantly changed as compared to healthy control tissues (*P*>0.05) (Figure 3A). No significant differences for the expression of PLK2 and CRK mRNAs were observed between active UC tissues and healthy controls (Figure 3B, C).

**Figure 4:**
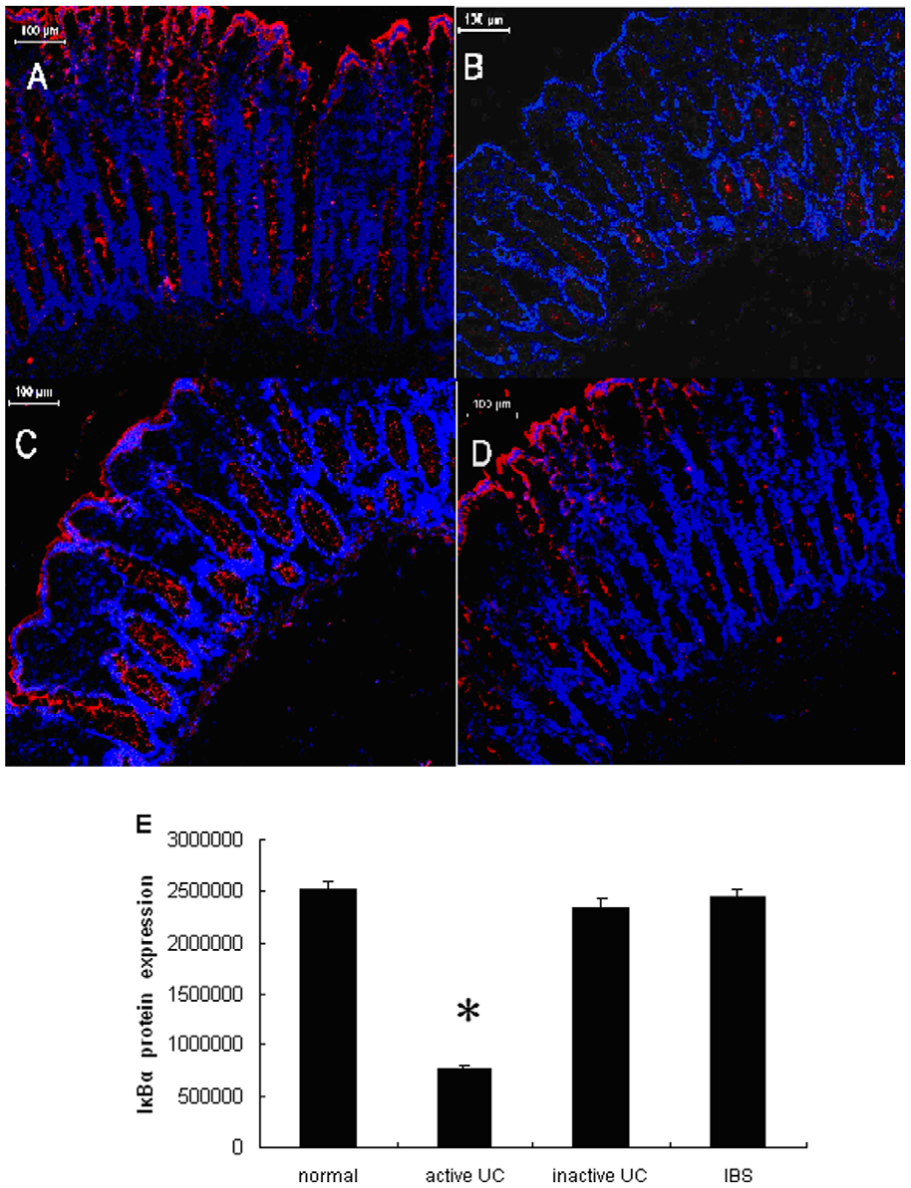
Localization of IκBα in human colon tissues. Representative frozen-sections were prepared from inflamed mucosa of an active UC patient (A), an inactive UC patient (B), a healthy control (C), and an IBS patient (D), and stained for IκBα by immunofluorescence. IκBα is significantly reduced in epithelial cells of active UC tissues, while it is detected in the epithelial cells of healthy controls, inactive UC and IBS tissues. Red, IκBα; blue, DAPI nuclear staining. Pictures were imaged at ×20 magnification on a Leica converted fluorescence microscope. (E) Fluorescence intensity of IκBα in each group was then calculated. Data is presented as mean ± SEM of 15 cases of healthy control, 12 cases of active UC, 10 cases of inactive UC and 15 cases of IBS (*P<0.05 compared to that of normal healthy individuals).

To determine whether the expression of IκBα protein was altered in colons with active UC, we further performed immunofluorescent staining on cryosections of sigmoid biopsies within each group. The staining results displayed that IκBα protein was clearly expressed in epithelial cells of normal colonic mucosa (Figure 4A and Figure S1). It was markedly decreased in active UC tissues, when compared to the other three groups (*P*<0.05) (Figure 4A, B and E). However, there was no differential expression of IκBα protein among inactive UC, IBS, and healthy control colon tissues (*P*>0.05) (Figure 4B, C, D and E).

### Inverse Correlation of IKBA and miR-126 Expression

**Figure 5:**
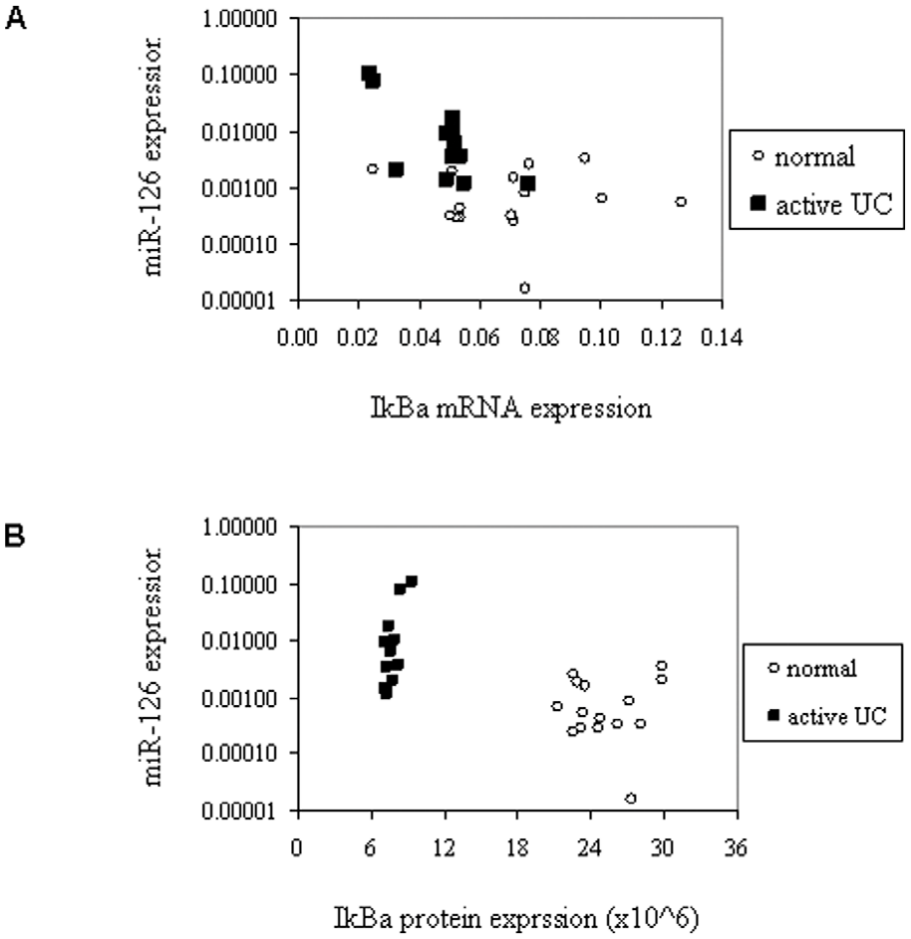
Correlation of IκBα expression and miR-126 expression in human colon tissues. (A) The expression of miR-126 and IKBA mRNA were detected by qRT-PCR in individual biopsy samples from normal healthy individuals (open circles, *n* = 15) and active UC (solid squares, *n* = 12), Pearson correlation analysis *r* = −0.466, *P*<0.01. (B) The expression patterns of miR-126 and IκBα protein (detected by immunofluorescence analysis as shown in Figure 4E) in samples from normal healthy individuals (open circles, *n* = 15) and active UC patients (solid squares, *n* = 12).

Our findings showed that miR-126 expression was significantly increased in active UC tissues as compared to healthy controls, inactive UC and IBS patients. In contrast, IκBα expression, mRNA and protein, were decreased markedly in active UC tissues. We found that the expression of miR-126 and IKBA mRNA were inversely correlated in active UC tissues and normal controls (Pearson correlation analysis *r* = −0.466, *P*<0.01) (Figure 5A). Such inverse correlation patterns were also present between miR-126 expression and IκBα protein level in active UC and normal control samples (Figure 5B). These results indicate that IκBα may be a target of miR-126 in the pathogenesis of active UC.

### Inhibition of IKBA Expression by miR-126

**Figure 6:**
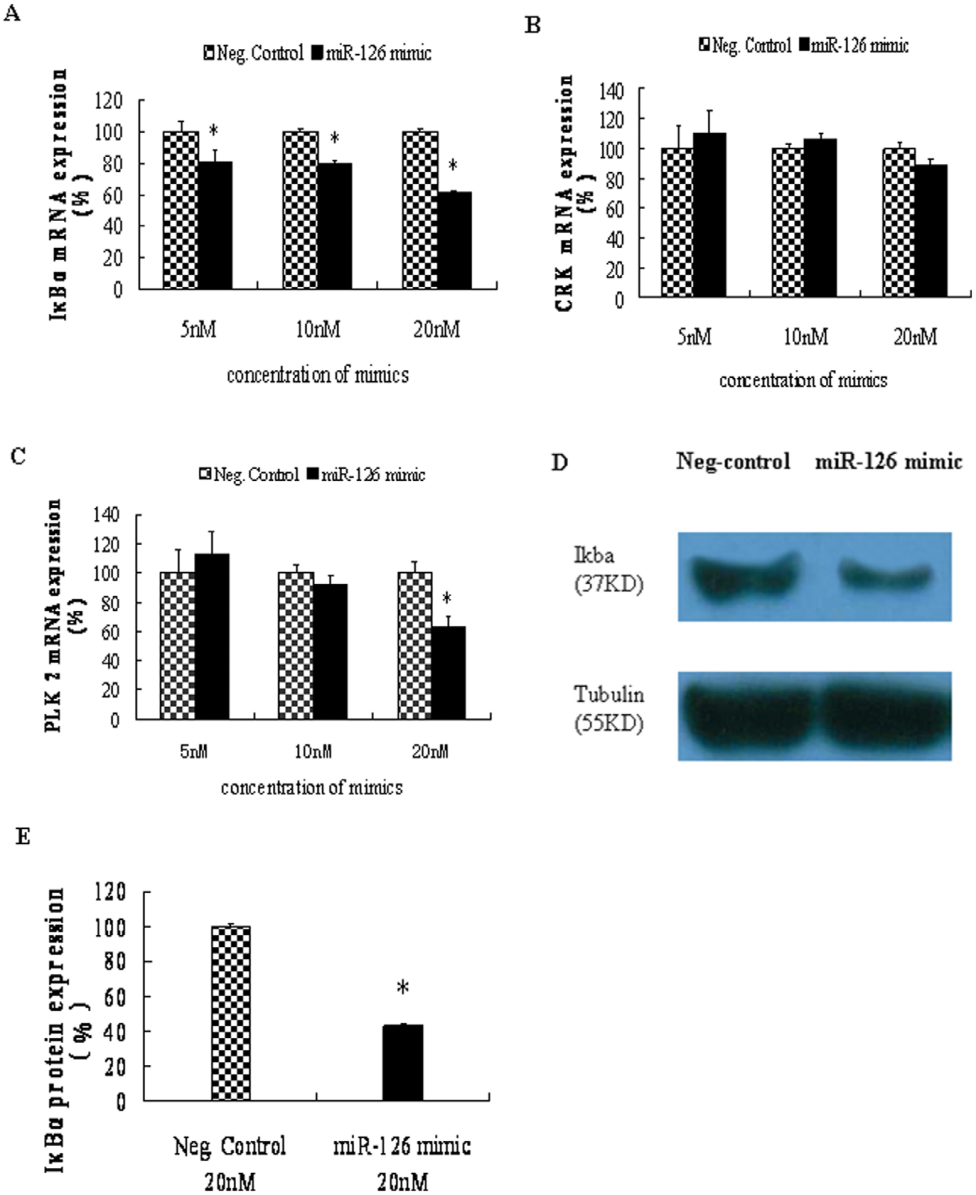
Effects of miR-126 mimic on expression of IKBA. HT29 cells were transfected with various amount miR-126 mimic or negative control mimic for 24 hours. Expression of (A) IKBA, (B) CRK and (C) PLK2 mRNA in total RNA of these cells were detected by qRT-PCR. (D) IκBα proteins of cells transfected with 20 nM of miR-126 mimic or the negative control were detected by Western Blot. Tubulin detection was served as loading reference. (E) The integral of optical density of (D) was measured using Quantity One program and normalized to corresponding density of Tubulin band. Data is presented as mean ± SEM of three independent experiments (*P<0.05, compared to that of negative control mimic treatment).

To test whether miR-126 regulates endogenous IκBα expression, the miR-126 mimic was transiently transfected into HT29 cells and then IKBA mRNA expression was assessed. IKBA mRNA expression was decreased by 20% and 40% in the cells transfected with 10 nM and 20 nM of miR-126 mimic, respectively (Figure 6A). However, the negative control mimic did not significantly reduce IKBA mRNA expression. In addition, we also checked the expression of CRK and PLK2 in these cells. The expression of CRK mRNA was not affected by the miR-126 mimic treatment, while PLK2 expression was reduced by about 35% in cells transfected with 20 nM of miR-126 mimic (Figure 6B, C). These results were consistent with previous reports [30]–[33].

Furthermore, we found that the IκBα protein expression in HT29 cells transfected with 20 nM miR-126 mimic, was significantly inhibited by 57% as detected by western blot analysis (Figure 6 D, E). Similar inhibitory effect of miR-126 mimic on IκBα protein expression was observed in the experiments using an additional colonic cell line HCT116 (Figure S2). These results suggest that miR-126 negatively regulates endogenous IκBα expression in colonic epithelia.

### Regulation of IKBA 3′-UTR by miR-126

**Figure 7:**
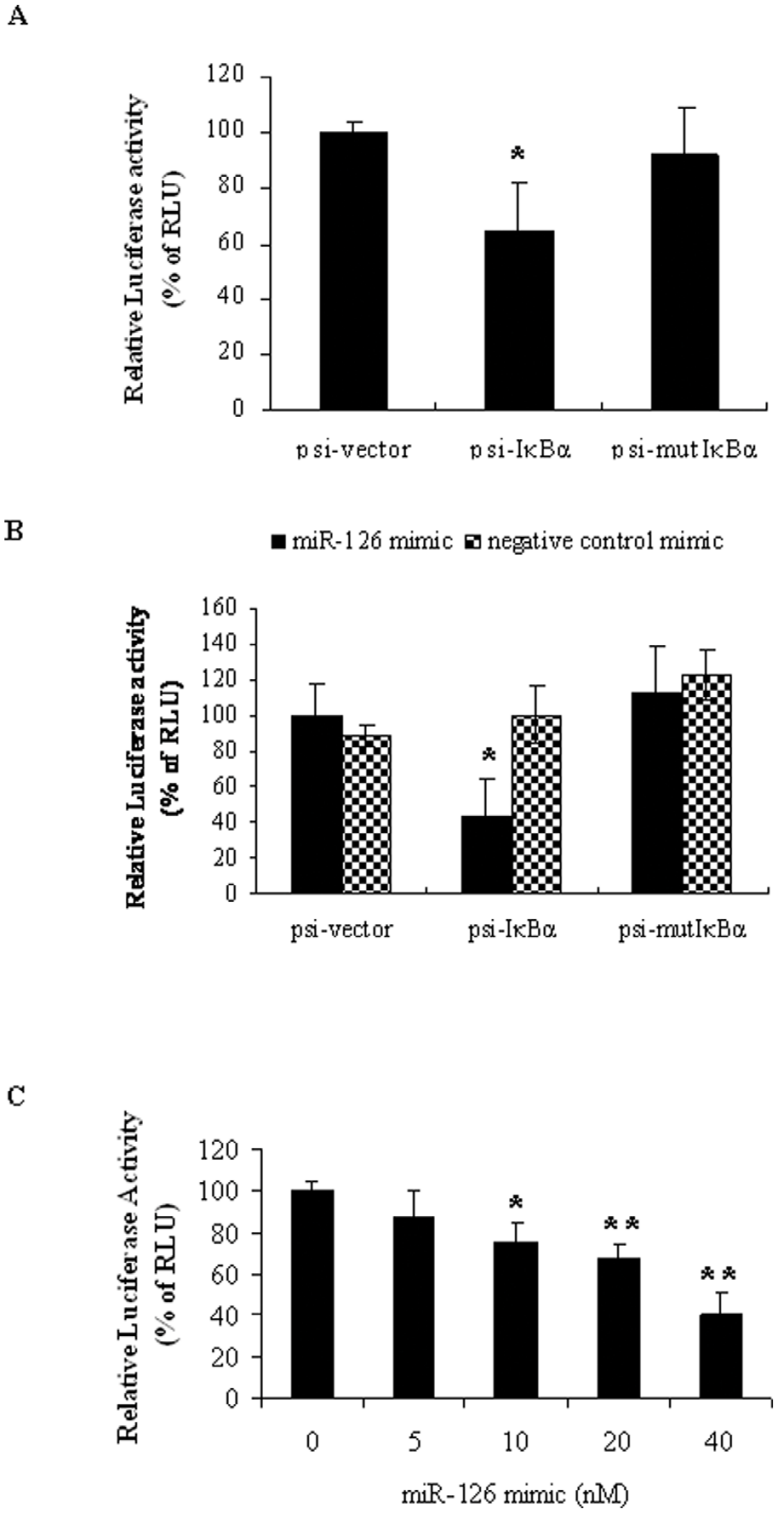
miR-126 targeting on 3′-UTR of IKBA. HT29 cells were transfected with either original dual luciferase reporter vector (psi-vector), or wild type miR-126 biding site of IKBA 3′-UTR construct (psi-IKBA) or mutated miR-126 biding site construct (psi-mutIKBA). Relative Renilla Luciferase activity (normalized to Firefly luciferase activity) was measured 24 hours post-transfecting. (A) Relative Renilla luciferase activity was reduced in cells transfected with wild type IKBA 3′-UTR construct (psi-IKBA) compared to that with the psi-vector (100 ng/well of a 24-well-plate). Relative Renilla luciferase activity was restored in cells transfected with mutant construct (psi-mutIKBA). (*P<0.05, compared to that of psi-vector). (B) Co-transfecting 10 nM miR-126 mimic with various luciferase reporter constructs (100 ng/well) into HT29 cells reduced the relative Renilla luciferase activity. (*P<0.05, compared to that of psi-vector) (C) miR-126 mimic showed dose-dependent effects on relative Renilla luciferase activity in cells transfected with wild type psi-IKBA construct (250 ng/well) for 24 hours. (*P<0.05, **P<0.01, compared to that without miR-126 mimic treatment). Data is presented as mean ± SEM of three independent experiments.

To determine whether miR-126 directly targets the 3′-UTR of IKBA mRNA, we transfected HT29 cells with an IKBA 3′-UTR luciferase reporter construct containing two wild type miR-126 putative binding sites (psi-IKBA) or a mutant construct bearing mutations on miR-126 binding sites (psi-mutIKBA). Compared to the parental luciferase reporter (psi-vector), the relative luciferase activity of the wild type psi-IKBA showed 35% reduction. Mutations of miR-126 binding sites in the IKBA 3′-UTR resulted in a restoration of luciferase activity to 92% of the parental reporter (*P*<0.01) (Figure 7A). These results indicate that endogenous miR-126 can regulate IκBα expression by directly targeting its 3′-UTR.

To further confirm the findings, the miR-126 mimic was co-transfected with the above luciferase reporter constructs into HT29 cells. The miR-126 mimic dramatically reduced (>55%) the luciferase activity of the wild type IKBA 3′-UTR reporter construct psi-IKBA, while a negative control mimic had no effect on luciferase activity in any group (Figure 7B). The inhibitory effect of miR-126 mimic on the wild type psi-IKBA also showed a dose-dependent response, even though a relatively high amount of the wild type psi-IKBA construct was used (Figure 7C).

However, miR-126 mimic did not reduce the luciferase activity of mutant construct psi-mutIKBA (Figure 7B), indicating its specific recognition effect. These results further indicate that miR-126 can regulate IκBα expression by directly targeting its 3′-UTR.

## Discussion

Numerous studies have demonstrated that different subtypes of IBD possess a distinct gene expression profile and identified a number of protein coding transcripts as molecular targets for pathogenesis of human IBD. Compared to protein coding genes, the roles of microRNAs, a subset of non-coding transcripts, in initiation and progression of IBD have not been extensively characterized. The involvement of microRNAs in pathological processes of IBD was first demonstrated in a recent study which revealed a strikingly unique signature of microRNA expression in different subtypes of IBD [18], later confirmed by several studies [19]–[27]. These findings suggest the critical roles of microRNA alterations in the development of chronic inflammatory processes of human IBD. From genome-wide microarray screening, miR-126 was previously identified as one of the up-regulated microRNAs detected in active UC patients in America [18]. In this study, we specifically analyzed alterations of miR-126 in a cohort of UC and IBS patients as well as healthy controls. Among three candidate microRNAs, we found that miR-126 and miR-21 were upregulated in colonic biopsies from patients with active UC, with 18- and 14.7-fold increases respectively. This finding was consistent with a previous study [18] and further suggests that miR-126 is a crucial molecular target for development of UC.

An inconsistent finding between previous genome-wide screening [18] and our current study was observed for miR-375 expression in UC patients, which may be attributed to patient populations with different ethnic and genetic backgrounds.

Our study provides the first evidence that upregulation of miR-126 is a novel mechanism in the development of UC. Although regulation of a number of molecular targets by miR-126 has been studied, involvement of pathways regulated by miR-126 in UC is completely unknown. In cancers, miR-126 has been found to possess paradoxical roles in different tumor types. miR-126 was found to target a tumor suppressor PLK2 and further inhibit apoptosis and increase the viability of acute myeloid leukemia cells [30]. On the other hand, miR-126 was shown to function as a tumor suppressor targeting on CRK in gastric cancer, mammary cancer and non-small cell lung carcinoma cell lines [31]–[33]. Recently, a few studies suggest that miR-126 is associated with human disorders other than cancers such as inflammation and fibrosis [34]–[36]. miR-126 was shown to down-regulate vascular cell adhesion molecule-1 (VCAM-1) expression and may provide additional control of vascular inflammation [34]. Selective blockade of miR-126 suppresses the asthmatic phenotype by diminishing TH2 responses [35]. These findings indicate that miR-126 regulates different molecular targets in different inflammatory processes.

To characterize the potential mechanism regulated by miR-126 in UC, we performed target scan analysis to identify the molecular targets of miR-126. We found that the 3′-UTR of IκBα mRNA possesses a miR-126 targeted sequence. IκBα is an important inhibitor for NF-κB. IκBα binds to the NF-κB complex and further inactivates NF-κB by retaining its cytoplasmic localization. The degradation of IκBα is in response to certain pro-inflammatory cytokine like TNF and results in dissociation of NF-κB from IκBα/p50/p65 complex. Consequentially NF-κB is translocated into the nucleus and activates NF-κB-mediated transcription of genes such as cytokines and chemokines. Constant activation of NF-κB activity has been a well-proven molecular event for human IBD including UC [37]–[39]. Several studies have established IκBα as a key inhibitor for NF-κB activity in IBD [37], [40]. Viral IκBα expression vectors have been shown to produce a preventive effect on NF-κB activation in mucosal macrophages and T lymphocytes as a treatment option in IBD [41]. Moreover, Teaflavin-3,3′-digallate which stabilizes IκBα from degradation, can inhibit the activity of NF-κB in colon tissues [42].

Here we demonstrated that IκBα is abundantly expressed in normal colonic epithelia, but significantly reduced in active UC tissues. More interestingly, we found a significant inverse correlation between miR-126 over-expression and down-regulation of IκBα in active UC tissues. We then demonstrated that endogenous miR-126 and exogenous miR-126 mimic can inhibit IκBα expression. Finally, mutating the miR-126 binding site in the IKBA 3′-UTR in a reporter construct resulted in the restoration of reporter gene expression. Taken together, our results imply that over-expression of miR-126 leads to NF-κB activation via down-regulation of IκBα, which may contribute to the pathogenesis of human UC.

NF-κB mediated pathways are complex biological processes and signaling components including IκBα in this important pathway are regulated by a number of molecules playing essential roles in different cellular functions. It was shown that TNF-α and interleukin-1(IL-1)activated IkappaB kinase-beta (IKK-beta) and further phosphorylate IκBα [43]. In addition, phosphatidylinositol (PI) 3′-kinase (PI3′K) can also directly lead tyrosine phosphorylation of IκBα in breast cancer cell models [44]. IκBα phosphoration consequentially results in its ubiquitination and degradation, which ultimately leads to activation of NF-κB signaling. Therefore, multiple molecular mechanisms in addition to miR-126 overexpression may potentially contribute to the regulation of IκBα expression in inflammatory processes of UC.

## Supporting Information

Figure S1 Localization of IκBα in human colon tissues. Representative frozen-section was prepared from a healthy control, and stained for IκBα by immunofluorescence. IκBα is detected in the epithelial cells of healthy colon tissues. Red, IκBα; blue, DAPI nuclear staining. Pictures were imaged at ×40 magnification on a Leica converted fluorescence microscope.
(TIF)

Figure S2 Effects of miR-126 mimic on expression of IKBA in HCT116. HCT116 cells were transfected with 20 uM of miR-126 mimic or negative control mimic for 24 hours. (A) Expression of IκBα proteins were detected by Western Blot. Tubulin detection was served as loading reference. (B) The integral of optical density of (A) was measured using Quantity One program and normalized to corresponding density of Tubulin band. Data is presented as mean ± SEM of three independent experiments (*P<0.05, compared to that of negative control mimic treatment).
(TIF)
