# Supplementary figures and images for: Up-Regulation of microRNA-126 May Contribute to Pathogenesis of Ulcerative Colitis via Regulating NF-kappaB Inhibitor IκBα
Source: PLoS One. 2012 Dec 28;7(12):e52782. doi: 10.1371/journal.pone.0052782 (PMC3532399; doi:10.1371/journal.pone.0052782)

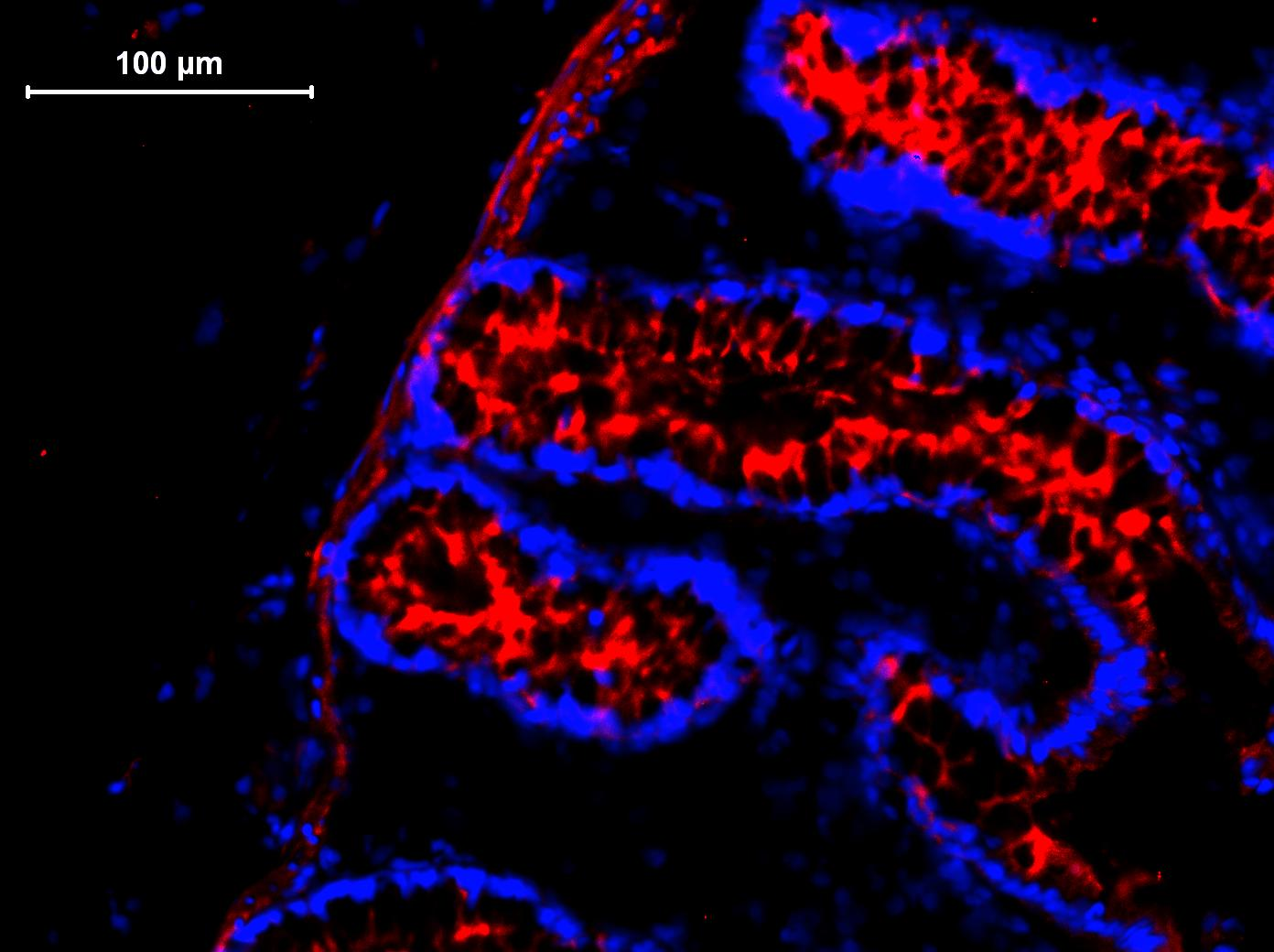

Supplement: Figure S1 — Localization of IκBα in human colon tissues. Representative frozen-section was prepared from a healthy control, and stained for IκBα by immunofluorescence. IκBα is detected in the epithelial cells of healthy colon tissues. Red, IκBα; blue, DAPI nuclear staining. Pictures were imaged at ×40 magnification on a Leica converted fluorescence microscope. (TIF) [file pone.0052782.s001.tif]

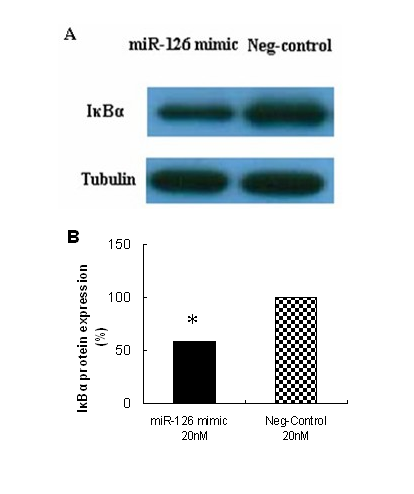

Supplement: Figure S2 — Effects of miR-126 mimic on expression of IKBA in HCT116. HCT116 cells were transfected with 20 uM of miR-126 mimic or negative control mimic for 24 hours. (A) Expression of IκBα proteins were detected by Western Blot. Tubulin detection was served as loading reference. (B) The integral of optical density of (A) was measured using Quantity One program and normalized to corresponding density of Tubulin band. Data is presented as mean ± SEM of three independent experiments (*P<0.05, compared to that of negative control mimic treatment). (TIF) [file pone.0052782.s002.tif]
